# Modulation of Central Synapses by Astrocyte-Released ATP and Postsynaptic P2X Receptors

**DOI:** 10.1155/2017/9454275

**Published:** 2017-08-06

**Authors:** Eric Boué-Grabot, Yuriy Pankratov

**Affiliations:** ^1^Institut des Maladies Neurodégénératives, University Bordeaux, UMR 5293, 33000 Bordeaux, France; ^2^Institut des Maladies Neurodégénératives, CNRS, UMR 5293, 33000 Bordeaux, France; ^3^School of Life Sciences, University of Warwick, Coventry, UK; ^4^School of Life Sciences, Immanuel Kant Baltic Federal University, Kaliningrad, Russia

## Abstract

Communication between neuronal and glial cells is important for neural plasticity. P2X receptors are ATP-gated cation channels widely expressed in the brain where they mediate action of extracellular ATP released by neurons and/or glia. Recent data show that postsynaptic P2X receptors underlie slow neuromodulatory actions rather than fast synaptic transmission at brain synapses. Here, we review these findings with a particular focus on the release of ATP by astrocytes and the diversity of postsynaptic P2X-mediated modulation of synaptic strength and plasticity in the CNS.

## 1. Introduction

Adenosine 5′-triphosphate (ATP) is arguably one of the most abundant molecules in living cells serving as universal energy “currency.” So, it is of no surprise that ATP is also widely utilized as an extracellular signaling molecule [1]. Extracellular ATP acts as an excitatory neurotransmitter in the brain, spinal cord, and peripheral nerve terminals [2–4]. Furthermore, ATP can act as a “gliotransmitter” transferring signals within glial networks or between neurons and glial cells [2–5]. The metabolic breakdown of extracellular ATP by ectonucleotidases is also a source of other nucleotides and adenosine. Adenosine acts as neurotransmitter and neuromodulator in the central nervous system (CNS) through activation of adenosine G protein-coupled receptors that are widely expressed in glia and neurons at both pre- and postsynaptic levels. Adenosine plays important physiological roles in the brain in health and diseases detailed in recent review articles [6–10]. The action of ATP as neuro- or gliotransmitter is mediated by a broad family of purinergic receptors expressed in neurons and glia. P2 receptors are classified into several subtypes of ligand-gated ion channels (P2X1–P2X7 subunits) and eight distinct G protein-coupled receptors (P2Y) which are both characterized by a variety of distinct properties and a broad range of ATP sensitivities ranging from nanomolar (P2Y receptor) to tenth micromolar (P2X) or millimolar for P2X7 [1, 11–14]. The seven P2X subunits share a unique and simple architecture with two hydrophobic membrane-spanning domains separated by a large extracellular domain and two intracellular termini. They assemble as homo- or heterotrimers to form diverse nonselective cation channels with distinct kinetics and pharmacological properties. All P2X subunits are expressed in neural cells in a heterogeneous manner through the brain regions, cell types, and subcellular compartments [15–17]. Consequently, the subunit composition of P2X receptors in most of central neurons is far from being characterized. Neuronal P2 receptors are expressed at pre- and postsynaptic loci [18]. Presynaptic P2 receptors play a critical role in the regulation of neurotransmitter release [10, 11] by contributing to the intracellular Ca^2+^ signaling [11, 13] by virtue of the high Ca^2+^ permeability (P2X) and ability to stimulate IP3-dependent Ca^2+^ release from endoplasmic reticulum (P2Y). These properties can underlie also an important role for postsynaptic P2X receptors in the modulation of synaptic activities highlighted relatively recently [12]. In this review, the recent knowledge on the role of postsynaptic P2X receptors focused on glia-neuron interactions is summarized.

## 2. Release of ATP by Glial Cells

An ability of astrocytes to release ATP has been suggested by studies showing the participation of ATP in the propagation of glial Ca^2+^ waves and the significant contribution of ATP and adenosine to the astroglia-driven modulation of neuronal activity and sleep homeostasis [3, 19–21]. A variety of molecular mechanisms of ATP release from astrocytes have been suggested, including exocytosis and concentration gradient-driven diffusion through large conductance channels such as gap junction hemichannels, anion channels, and dilated P2X7 receptors [3, 5, 21].

In addition to astrocytes, a significant amount of extracellular ATP can be released from microglia, in particular during neuroinflammation [2, 22–24]. Microglia-derived ATP has been reported to activate P2X receptors in the hippocampal and spinal cord neurons [22–24].

From the early days of research into glial-neuron interaction, a concept of fast vesicular release of chemical transmitters, including ATP, from astrocytes attracted a big attention and was embedded in the popular concept of tripartite synapse [25] which had implied the equal importance of astrocytes for synaptic physiology. Indeed, there is a large body of evidence that the release of ATP from astrocytes may share common mechanisms of vesicular neurotransmitter release such as a dependence on the proton gradient, vesicular transporters, and SNARE proteins and intracellular Ca^2^^+^ elevation [20, 26–29]. There are also accumulating reports of physiological roles for SNARE-dependent glial exocytosis [19, 21, 28]. In particular, exocytosis of ATP followed by its conversion to adenosine has been implicated into the regulation of LTP in the the hippocampus and sleep homeostasis in the hypothalamus [21]. The key element of latter works was the development of dnSNARE transgenic mice with inducible inhibition of exocytosis selectively in astrocytes [21].

Yet, the physiological relevance of vesicular release of gliotransmitters is intensively debated [26, 27]. This debate has been fuelled by an argument that the bulk of evidence supporting the SNARE-dependent release of gliotransmitters was obtained mostly in cell cultures where functional properties of astrocytes can be dramatically altered by various artefacts [27]. Until recently, there were mainly circumstantial evidence of Ca^2+^-dependent release of ATP from glial cells in brain slice based on the inhibitory action of P2X receptor antagonists on glia-driven modulation of synaptic transmission and plasticity [19, 30, 31]. A definitive evidence of vesicular release of ATP from astrocytes in situ, based on several experimental approaches, has been demonstrated only recently [28, 32, 33]. These works have demonstrated that quantal release of ATP from cortical astrocytes can be triggered by the cytosolic Ca^2+^ elevation, attainable in physiological conditions [28, 32, 33] and can be inhibited by the intracellular perfusion of astrocytes with light chain of tetanus toxin or inhibitors of vesicular nucleotide transporters. The release of ATP was impaired in the transgenic mice expressing dnSNARE protein selectively in astroglial cells [28, 33]. One should emphasize that this effect could hardly be attributed to the allegedly “leaky” expression [34] of dnSNARE transgene in neurons since there was no deficit in the GABAergic and glutamatergic synaptic transmission [28, 33]. Also, the lack of neuronal expression of dnSNARE has been recently verified by other labs [35, 36]. The release of ATP from cortical astrocytes can occur, most likely, from synaptic-like microvesicles and lysosomes [28]. Importantly, these works also provide a direct demonstration of capability of astrocyte-derived ATP to activated P2X receptors in neurons, most likely located at the extrasynaptic sites [28].

The above works also reported a presence of nonvesicular release of ATP from astrocytes which is in agreement with previous data suggesting a participation of connexin hemichannels and/or volume-sensitive channels in glia release of ATP [3, 30, 37]. However, the contribution of nonvesicular mechanisms into the release of ATP from cortical astrocytes at physiological conditions was not large and the dominant role was played by vesicular mechanisms [28]. The major contribution of vesicular mechanisms into the release of ATP has also been reported for brainstem astrocytes in vivo [19]. The notion of the coexistence of vesicular and nonvesicular mechanisms of ATP release and the dominant role of vesicular pathway are in line with previous results obtained in the spinal cord astrocytes [37]. The latter work showed that fast initial astroglial exocytosis of ATP triggered a robust, but slow, secondary release of ATP through the pannexin and connexin hemichannels [37]. One should also note that the data supporting a major role for nonvesicular mechanisms of ATP release in the brain were often reported by works aimed to study the excitotoxicity of ATP after ischemic tissue damage or during neurological disorders [2, 3, 38]. Thus, a consensus view on the mechanism of glial ATP release might be a participation of both exocytosis- and channel-dependent pathways, with the relative contributions former being smaller at physiological conditions but increasing in the context of brain pathologies. It is also feasible that relative contribution of vesicular/nonvesicular mechanisms into gliotransmitter release can be region-specific as many other functions of astrocytes. There is also evidence that the release of ATP from astrocytes can undergo significant age-related changes [39]. Whatever is a mechanism, the release of ATP represents a powerful pathway of glia-neuron interaction which has already been implicated into a variety of physiological functions, including breathing control [19], slow neuromodulation [30, 31, 40], and modulation of sleep homeostasis [21] (after conversion into adenosine). Yet, the most intriguing and frequently overlooked role for glia-derived ATP is the regulation of synaptic strength, which will be discussed in detail below.

## 3. Neuromodulatory Action of Postsynaptic P2X Receptors in the CNS

ATP showed to be released by exocytosis from the nerve terminal and acts as a fast synaptic neurotransmitter in the nervous system though activation of postsynaptic P2X receptors [41–46]. If there is robust evidence of fast transmission mediated by ATP and P2X receptors in the peripheral nervous system such as sympathetic neurons, at neuromuscular junction or in myenteric nervous system, such a form of ATP-mediated fast synaptic transmission is more rarely observed in the CNS (see for review [11]). Following the first evidence for fast excitatory ATP postsynaptic currents recorded in the medial habenula [41, 42], fast ATP synaptic transmission mediated by activation of postsynaptic P2X receptors was also reported in several other brain regions such as the *locus coeruleus*, the hippocampus, the hypothalamus, and the cortex as well as in the spinal cord [5, 43, 44, 46, 47]. However, evoked P2X-mediated postsynaptic currents required generally strong electrical stimulation of afferent fibers and are of weak amplitude [32, 45]. To date, there is no example of action potential firing induced by synaptic release of ATP and activation of P2X receptors in the CNS. In addition, rare spontaneous or miniature P2X-mediated excitatory postsynaptic currents were recorded at central synapses conveying the idea that ATP and postsynaptic P2X receptors do not act primarily as fast excitatory neurotransmitter at brain synapses [12] and, thus, despite the detection of several P2X subunits at the postsynaptic sites of glutamatergic synapses [15]. Several studies suggested that postsynaptic P2X receptors modulate fast synaptic responses mediated by other fast neurotransmitters (see for review [12, 48]). Recent works have showed that ATP released by glial cells and/or by the presynaptic terminals acting on postsynaptic P2X receptors have various and profound effects on synaptic efficacy and plasticity at both the excitatory and inhibitory synapses in several brain areas [28, 30–32, 40, 49]. There is also evidence of abundant expression of various P2X receptor subtypes at presynaptic *loci* [15] and presynaptic effects of P2X and P2Y receptors on the release of glutamate, GABA, and other neurotransmitters in different brain regions and the spinal cord [1, 10, 11, 15, 50–52]. These results gave rise to the hypothesis that the main role for ATP and P2X-mediated signaling in CNS is neuromodulation rather than fast neurotransmission [12, 52].

Although the presynaptic action of the P2 receptors on the release of various neurotransmitters have been studied for last two decades (see for review [10, 11]), the notion of physiological importance of the postsynaptic purinergic modulation has been highlighted relatively recently [12]. Several arguments were already in favor of the modulatory action of ATP released from nerve terminals and postsynaptic P2X receptors. The first is that no brain synapses to date used solely ATP as excitatory neurotransmitter but ATP is frequently coreleased from the same terminals with other transmitters such as glutamate or *γ*-amino-butyric acid (GABA) [32, 43, 44, 47, 53]. Consistent with this idea, electron microscopy indicated that P2X2, P2X4, and P2X6, the predominant fast purinoceptors expressed in the majority of brain neurons, are postsynaptically located and mainly localized at the edge of the postsynaptic density synapses in the peri- and extrasynaptic space [16] at least at the basal levels of neuronal activity. In contrast, AMPAR or GABA_A_R which mediate, respectively, the rapid excitatory and inhibitory synaptic transmission are concentrated at the synapses facing the site of neurotransmitter release from the presynaptic terminal [54, 55]. Single-molecule imaging methods allow now the visualization of the lateral movement of receptors in the postsynaptic membrane. They have revealed that AMPAR and GABA_A_R permanently exchange between synaptic and extrasynaptic locations and alternate between periods of lateral mobility at extrasynaptic sites, and periods of immobility at synapses [56]. In contrast, single particle tracking of P2X2 or P2X4 receptors has shown that they are all very mobile and that their diffusion can be facilitated upon their activation by ATP [57]. In transfected neurons, P2X2 receptors moved within the membrane of dendrites and at extrasynaptic sites. In addition, these data showed that P2X2 receptors rarely enter into the glutamatergic synapse suggesting that P2X receptors are excluded from the postsynaptic density synapses reinforcing the electron microscopy data [58].

The lack of detectable P2X-mediated synaptic current is also consistent with the fact that the surface trafficking of P2X4 receptors is highly regulated. P2X4 receptors are constitutively internalized by dynamin-dependent endocytosis [59, 60] through interaction between the adaptor protein AP2 and a novel noncanonical endocytosis motif present exclusively in P2X4 subtype [59, 61, 62]. As a result, P2X4-containing receptors are retained predominantly in intracellular pools, limiting their putative implication in ATP signaling originating from either neuron or glia in normal conditions. However, intracellular pools of P2X4 receptors that are resistant to lysosomal degradation can be rapidly trafficked to the surface membrane increasing the surface number of functional P2X4 receptors [63]. Apart from P2X2, P2X4, and P2X6 subtypes, the presence of other subtypes, such as P2X1 or P2X3, in the central synapses has been suggested based both of the electrophysiological/pharmacological data and colocalisation with synaptic markers [15, 44, 45]; the presence of heteromeric P2X1/4 and P2X2/3 also cannot be excluded. Since P2X1 and P2X3 receptors are prone to the fast desensitisation, internalisation, and lateral mobility [1, 64], the subtype composition and overall density of P2X receptors at the given synapse may undergo substantial variations, very likely in the activity-dependent manner. Interestingly, a recent study of P2X receptor-mediated signaling in individual cortical synapses suggested that ATP released either from synaptic terminals or from astrocytes can upregulate the trafficking of postsynaptic P2X receptors via different Ca^2+^-dependent mechanisms [32].

This is likely to be important because an upregulation of surface P2X receptors (in particular P2X4 subtype) was observed in various pathophysiological contexts such as ischemia, chronic pain [23], or neurodegenerative diseases such as amyotrophic lateral sclerosis (ALS) or Alzheimer's disease (AD) [38, 65–67]. So, excessive release of ATP from various sources, including astrocytes and microglia, can confer a particular importance of the neuromodulatory action of P2X receptors in pathological conditions.

## 4. Modulation of Glutamatergic Excitatory Synapse by P2X Receptors

NMDAR and AMPAR have critical roles in excitatory synaptic transmission and plasticity in the CNS [68]. Synaptic strength is thought to be determined in part through the activity-dependent insertion and removal of synaptic AMPAR which lead to long-term potentiation (LTP) and long-term depression (LTD), that are widely recognized to underlie cognitive functions such as learning and memory [68–70]. AMPARs are tetrameric complexes composed of GluA1-A4 subunits [71]. The lateral diffusion and the intracellular trafficking (endocytosis/exocytosis) are regulated by subunit-specific AMPAR-interacting proteins as well as by various posttranslational modifications on their intracellular domains [72, 73]. The bidirectional fast changes in the number of synaptic AMPARs are mainly triggered by activation of NMDAR or mGluR during synaptic plasticity.

### 4.1. Direct Modulation of AMPAR and Glutamatergic Synapse Efficacy by Glial ATP

**Figure 1:**
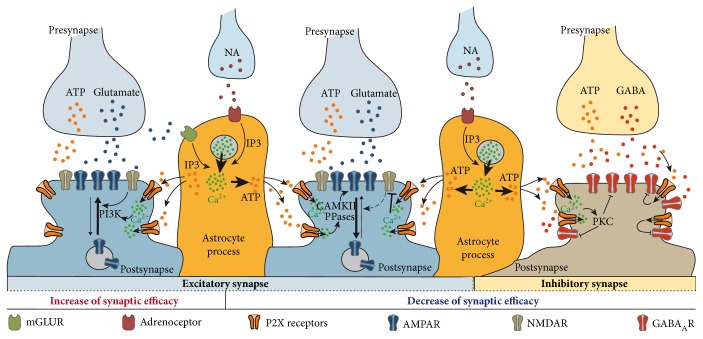
Summary of the postsynaptic P2X-mediated modulation of synaptic strength and plasticity following the release of ATP by astrocytes or neurons. Glutamate and noradrenaline acting on astrocytes cause the release of glial ATP. Activation of postsynaptic P2X receptors at glutamatergic synapses (blue) can trigger either a PIK3-dependent insertion of AMPAR leading to the increase of synaptic strength and synapse scaling (left) or a CaMKII-dependent internalization of AMPAR leading to a P2X-dependent synaptic depression (middle). P2X receptors can also cause an alteration of glutamatergic synapse plasticity by inhibiting NMDA function by interfering on PSD-95/NMDAR complex. At inhibitory synapses (brown), glial or neuronal ATP decreases GABAergic synapse efficacy by direct or indirect alteration of GABA_A_ (right).

Two noteworthy studies showed that direct activation of postsynaptic P2X receptors by endogenous ATP plays also a crucial role in glutamatergic synaptic transmission [30, 40]. These two works provide strong evidence that postsynaptic P2X receptors activated by ATP released from astrocytes resulted in enduring changes of glutamatergic synaptic efficacy in distinct brain areas. The first work showed in the paraventricular nucleus of the hypothalamus, a long lasting increase in the amplitude of AMPAR-mediated mEPSC in response to brief application of noradrenaline (NA) that requires the release of ATP from glial cells [30]. It has been demonstrated that NA acted on astrocytic *α*1-adrenoceptors and triggered the release of ATP by astrocytes. In addition, the pharmacological blockade by fluoroacetate (FAC) of the glial metabolism or physiological decrease by dehydration of the astrocytic coverage of the synapses onto magnocellular neurons in the hypothalamic neurosecretory system suppressed the increase in synaptic strength. The increase of AMPAR-mediated mEPSC is mediated by P2X receptors' activation and calcium influx and phosphatidylinositol 3-kinase (PI3K) resulting likely in the insertion of new AMPAR at the synapse. The effect of NA on the AMPAR-mediated mEPSCs was mimicked by ATP or benzoyl ATP, a more stable analogue of ATP, and blocked in the presence of brilliant blue G (BBG) in favor of the involvement of P2X7 receptors [30]. P2X7 subunit is possibly expressed in the magnocellular neurons of the hypothalamus with several other subunits [17]; but P2X7 is rarely expressed in neurons suggesting that this mechanism could be restricted to specific neuronal populations. It was showed later that P2X2 and P2X5 formed a heteromeric P2X receptor with an apparent affinity for ATP similar to P2X2 (~10 *μ*M) and P2X7-like functional properties [74]. This suggests that such form of plasticity could be mediated by more classical neuronal P2X receptors and consequently more broadly expressed in the brain (Figure 1).

The second work showed that activation of postsynaptic P2X (likely P2X2) receptors by NA-dependent glial release of endogenous ATP changed the glutamatergic synapses efficacy in the hippocampus but in an opposite way [40]. Brief application of ATP or NA-induced glial ATP release caused an enduring and slow decrease of AMPAR-mediated mEPSCs in hippocampal cultures. The effects of both ATP and NA were blocked by PPADS, a P2X antagonist, and by FAC that impairs astrocyte function. The same observation was reproduced on hippocampal brain slices where ATP and NA caused a depression of field excitatory postsynaptic potentials (fEPSPs) recorded in CA1 region that was also blocked by PPADS or FAC. By complementary approaches, including recordings and biochemistry from heterologous *Xenopus* oocytes expressing mammalian P2X2 or P2X4 with AMPAR, and high-resolution imaging from hippocampal neurons, Pougnet and colleagues demonstrated that activation of P2X2 or P2X4 receptors and calcium influx though the opening of P2X channels triggered dynamin-dependent internalization of GluA1 or GluA1/A2 AMPARs leading to reduced surface AMPARs in dendrites and at synapses [40].

Although no fast ATP synaptic transmission was detected in both brain structures, these studies showed that ATP released by astrocytes increased the efficacy of glutamatergic synaptic transmission in paraventricular nucleus and depressed synaptic efficacy in the CA1 region of the hippocampus [30, 40]. This raised the possibility that astrocyte-derived ATP and P2X receptors may bidirectionally change the glutamatergic synapse efficacy in the brain.

Gordon and colleagues have also established that the ATP signaling mechanism is engaged during an afferent activity [31]. The activity-dependent release of NA or glutamate by nerve terminals activates astrocytic mGluR or adrenoceptors (Figure 1). The subsequent increase of intracellular calcium in astrocyte leads to the release of ATP by astrocytes and consequently activation of postsynaptic P2X receptors of a large group of synapses covered by the astrocyte. Astrocytic activation may spread within astrocytes resulting in multiplicative scaling of all synapses in MCNs. Thus, although not contributing to the synaptic transmission, astrocyte-derived ATP signaling is likely exerting strong neuromodulation during physiological neuronal activation [31]. In addition, recent studies [75, 76] showed that startle and arousal responses in vivo are followed by NA-induced Ca^2+^ signaling in cortical astrocytes raising also the possibility that P2X receptors may contribute to the regulation of these behaviours [29].

The P2X-induced bidirectional changes in the efficacy of glutamatergic transmission induce insertion and removal of AMPAR at the synapses [30, 40]. P2X-mediated insertion of AMPAR in MCNs is linked to PIK3 kinase [30] that is also crucial for NMDA-dependent AMPAR insertion and LTP [69] suggesting that P2X receptors may serve as a “surrogate” for NMDAR [30]. In the hippocampus, P2X- and NMDA-induced depression in the CA1 region were additive, suggesting that both receptors are involved in distinct mechanisms leading to the removal of AMPAR from the synapses [40]. This assumption was confirmed recently by a work showing that P2X-mediated internalization of AMPAR is associated to the phosphorylation level of two calcium/calmodulin-dependent protein kinase II (CaMKII) phosphorylation sites S567 and S831 located in the cytoplasmic Loop1 and C-terminal tail of GluA1 subunits [49]. This work revealed also that P2X-mediated AMPAR inhibition is dependent on the subunit composition of AMPAR. GluA1 or GluA1/A2 are regulated by P2X activation while GluA3 homomers are insensitive and their presence in heteromers alters P2X-mediated inhibition. In addition, P2X-mediated depression in CA1 P2X-induced depression in hippocampal slices produces a dephosphorylation of the GluA1 subunit at S567, contrary to NMDAR-mediated LTD [49] (Figure 1).

Opposite effects of P2X-mediated changes of glutamatergic synapses' efficacy were both triggered by the Ca^2^ influx through the opening of P2X channels that activate distinct intracellular pathways regulating AMPAR trafficking. Does postsynaptic P2X receptors may bidirectionally change the efficacy of a synapse depending on astrocytic release of ATP, the dynamic of the Ca^2+^ influx though P2X receptors, or the nature of AMPAR? Such possibility will be of interest but requires additional studies since opposite effects were observed in distinct neuronal population recruiting very likely distinct P2X receptors.

### 4.2. Modulation of NMDAR-Dependent Plasticity

The NMDAR-dependent LTP in hippocampal CA1 neurons was shown to be modulated by P2X manipulation. Pharmacological blockade of P2X facilitated the induction of NMDA-dependent LTP [46, 77] at lower frequencies of action potential firing in Schaffer collateral axons suggesting that P2X receptor activation reduced NMDA-dependent LTP at low frequency stimulation [46, 77]. In contrast, a study using P2X4 knockout mice revealed that LTP in CA1 neurons was slightly reduced compared to that in wild-type mice [78]. In addition, ivermectin, a positive modulator of P2X4 function or trafficking [79, 80], enhanced LTP in the wild-type but not in P2X4 knock-out mice indicating a facilitatory action of P2X4 for hippocampal LTP [78]. No change of the fast excitatory synaptic transmission between P2X4 knockout and wild-type mice confirmed the absence of contribution of P2X4 in synaptic transmission, and the results suggested that P2X4 receptors regulated NR2B content within synaptic NMDAR [81].

Although controversial, altogether, these data argue in favor of the neuromodulatory action of ATP signaling rather than in fast synaptic transmission but also indicate that function of P2X receptors on NMDAR-dependent plasticity was far to be understood. Recently, a study dissected the role of synaptic and glial release of ATP in individual cortical synapses [32]. This work provided the direct evidence that ATP released from the individual excitatory synapses and/or glial cells can activate P2X receptors in the neocortical neurons. Moreover, the release of ATP from glial astrocytes turned out to trigger a recruitment of P2X receptors into synapses via Ca^2+^-dependent mechanism. Dependence of P2X recruitment to synapses on glial activity may explain why, despite evidence of widespread expression of P2X receptors in central neurons, the purinergic components of fast EPSCs are rarely observed and often require stronger stimulation to evoke [12, 45].

So, glial and neuronal release of ATP can converge to activation of postsynaptic P2X which, in turn, can cause a significant decrease in the synaptic currents mediated by NMDAR (Figure 1). This effect was underlined by the Ca^2+^-dependent dephosphorylation and interactions between NMDARs and calcineurin within PSD-95 protein complex [32, 82]. P2X-mediated downregulation of NMDA receptors was inhibited in the mutant mice, lacking PSD-95/Dlg4 domain (PSD-95 mutants) and the P2X4 knock-out mice [32]. As one might expect, the downregulation of NMDAR-mediated synaptic signaling had a strong impact on long-term synaptic plasticity. Impairment of purinergic modulation of NMDAR in the PSD-95 mutant mice led to substantial upregulation of the long-term synaptic plasticity in the neocortex which is manifested in the decrease in the threshold of LTP induction and increase in the net magnitude of LTP. These results are in line with the previous observation of facilitation of LTP induction in CA1 neurons after the inhibition of P2X receptors [46] and previously reported increase in the extent of LTP in PSD-95 mutant mice [82]. One should note that purinergic downregulation of NMDARs was not mediated solely by P2X4; contribution of other P2X subtypes was suggested by pharmacological data [32, 46]. The P2Y receptors could bring some, albeit moderate, contribution in the downregulation of NMDARs since this effect was significantly reduced after the removal of extracellular Ca^2+^ and could be mimicked to a large extent by application *α*,*β*-meATP—a selective P2X agonist [32, 43].

Interestingly, attenuation of purinergic modulation of NMDARs in P2X4 KO mice also shifted the threshold of LTP induction towards weaker stimuli but reduced the net LTP amplitude. In general, such reduction of the LTP amplitude in the neocortex agrees with previously reported decrease of LTP magnitude in the CA1 neurons of P2X4 KO mice [78]. The diversity of effects exerted by P2X receptors on the LTP suggests an involvement, in addition to inactivation of NMDARs, another molecular cascades such as the abovementioned bidirectional modulation of activity and trafficking of AMPARs and the downregulation of GABA receptors (see below).

Combined, these findings suggest an important role for the interaction between postsynaptic P2X and NMDARs in the regulation of synaptic strength. Coupling of NMDA receptors to PSD-95 and multiprotein complex is essential for the bidirectional modulation of synaptic strength and, therefore, for proper function of synaptic networks underlying learning and memory [82]. It has been shown previously that overenhancement of LTP in the PSD-95 KO mutants is accompanied by severe impairment of different forms of learning [82, 83]. It becomes evident now that this important pathway of control of NMDA receptor activity can be exploited by postsynaptic P2X receptors activated by glia-derived ATP. Such “premoderation” of plasticity of excitatory synapses can help to avoid “unwanted” or “excessive” LTP, that is, reduce number of errors. This mechanism can also increase a dynamic range of potentiation by presetting the lower baseline level of the synaptic activity. The P2X-mediated endocytosis of AMPAR [12, 40] can also contribute to this mechanism.

## 5. Modulation of GABAergic Inhibitory Synapse

The modulation of GABAergic system by P2X receptors was first observed by the action of presynaptic P2X receptors located on GABAergic terminals in the retina, spinal cord, or in midbrain where they regulate the release of GABA [50, 84, 85]. Direct modulation by postsynaptic P2X receptors of GABAergic inhibitory synaptic transmission was first identified in the ventromedial nucleus of the hypothalamus [60, 86]. Blocking the constitutive endocytosis of P2X4 receptors expressed in steroidogenic factor 1 (SF-1) positive neurons increased responses evoked by exogenous application of ATP but did not reveal fast ATP synaptic transmission. However, blocking P2X4 endocytosis depressed inhibitory GABA-mediated postsynaptic currents and consequently enhanced neuronal excitability of SF-1 neurons. GABA current inhibition was independent of ion flow through the P2X receptors and dependent on a direct interaction between P2X4 and GABA_A_Rs [60]. In addition, disruption of the physical coupling between P2X4 and GABA_A_ receptors using competitive peptides corresponding to the interaction domain identified in the C-terminal tail of P2X4 abolished P2X4 receptor-mediated GABAergic depression in SF-1 GFP-positive neurons. Molecular and functional cross-inhibition was also observed between P2X2 or P2X3 and nicotinic, GABA_A_ and 5-HT3 receptors in different native or recombinant cells [87–93]. Therefore, we should consider the possibility that P2X receptors, although almost silent during fast transmission, may be activated by ambient ATP and exert direct action on various other neurotransmitter receptors at central synapses (Figure 1).

Indeed, a recent study demonstrated that ATP released from cortical astrocytes modulated phasic and tonic GABAergic inhibition [28]. In this case, the downregulation of GABA_A_R was mediated by the calcium entry through the opening of P2X receptors. The downregulation of both tonic and phasic GABAergic transmission relied on the phosphorylation by PKC and was significantly reduced in the P2X4 KO and dnSNARE mice. Since phasic and tonic GABA conductances can influence membrane depolarization, one may expect that the downregulation of GABA receptors by astrocyte-derived ATP can be implicated in the glial modulation of the long-term potentiation. Indeed, glial exocytosis of ATP has been recently reported to facilitate the induction of LTP in neocortical neurons [33, 94]. This effect is dependent on the activity of P2X receptors [33, 94] and can be mimicked by attenuation of GABAergic inhibition by GABA_A_R antagonists [33]. Most likely, P2X-mediated attenuation of GABAergic inhibition facilitates LTP induction via increasing the depolarization of postsynaptic neurons and, therefore, alleviating the Mg^2+^ block of NMDA receptors (Figure 1).

## 6. Concluding Remarks

In the ongoing debate on physiological significance of purinergic gliotransmission, the leading role is usually assigned to adenosine receptors which can modulate synaptic transmission and plasticity via presynaptic and postsynaptic mechanisms [2, 3, 20, 21, 26, 95]. In the context of glia-neuron communications, ATP is often considered merely as a precursor of adenosine [3, 20, 26, 96]. However, the recent data discussed in this review highlighted new roles for ATP as a gliotransmitter and importance of P2X receptors for the modulation of synaptic transmission at postsynaptic sites. Furthermore, some of these data have revealed the dynamic cooperation between synaptic and glial release of ATP in the regulation of synaptic strength. Importantly, ATP released from astrocytes can facilitate a recruitment of P2X receptors into excitatory synapses by Ca^2+^-dependent mechanism [32] thereby acting upstream of synaptic purinergic signaling. This may underlie a particular physiological relevance of glial release of ATP.

Taken together, the recent data obtained (by different groups) in the different brain regions and physiological/pathological context suggest the existence of complex pathways of glia-derived purinergic regulation of synaptic transmission at postsynaptic *loci*. These pathways, which include the bidirectional modulation of AMPAR-mediated synaptic transmission, inactivation of NMDAR, and downregulation of GABAergic inhibition, rely on different intracellular signaling cascades. So, the prevalence of one or another pathway will be affected by the level expression of specific proteins (i.e., P2X of different subtypes, PKC, PI3K, CaMKII, and calcineurin) in the particular neuron of particular brain region. At the same time, the release of ATP from astrocytes can be activated virtually by any source of cytosolic Ca^2+^ elevation [28, 30, 32, 33, 40], which reaches certain threshold. Hence, the purinergic gliotransmission may have a different outcome on synaptic plasticity (potentiation versus depression) depending on the physiological context and activity of local networks.

There is growing recognition of the importance of neuronal-glial networks for coordination of metabolism and information processing in the brain, both in health and disease [20, 26, 96–98]. The capability of purinoreceptors, activated by glia-derived ATP, to trigger various Ca^2+^-dependent intracellular cascades, can make them very important for such coordination. As discussed above, purinergic astroglial modulation of synaptic transmission has many peculiar features, such as slower time-scale, the dependence on the activity of surrounding synapses, and ability to change the balance between excitatory and inhibitory synaptic inputs. These features can underlie an importance of astroglial release of ATP for heterosynaptic metaplasticity [96].

One could argue that coordination of activity over large astroglia syncytium might compromise the synapse specificity of glia-neuron communication, and therefore, the modulatory action of gliotransmitters, in particular ATP, would lack a selectivity needed for Hebbian plasticity. However, synaptic plasticity and memory require cooperation between many cellular and molecular mechanisms within neuronal ensembles, in particular for storage and retrieval of memory [99, 100]. There is growing consensus that memory and learning rely on distributed spatial and temporal representations of events overlaid in a distributed manner within a common neuronal circuit [100]. So, it is possible that glia-derived “preconditioning” of excitatory and inhibitory synapses within neuronal ensemble, for example, via P2X receptor-linked mechanisms discussed above, can affect the possibility of associations between stimuli/events. Also, there is emerging evidence supporting the physiological importance of fast local Ca^2+^ events in the astrocytic microdomains [101]. Potentially, astrocytes are capable of localized interaction with individual synapses. The ability of hippocampal and cortical astrocytes to generate Ca^2+^ elevations in response to activation of single synapse or single axon has been reported [32, 102]. The activity of astrocytes can also be input-specific [103]. Thus, it is conceivable that local Ca^2+^ transients in tiny astrocytic processes can trigger spatially constrained release of gliotransmitters, for example, ATP, and selectively affect the activity of an individual synapse. A putative physiological implication of such local glia-neuron communications is yet to be investigated.

Interestingly, several works have shown that astroglial *α*1-adrenoreceptors can efficiently activate the release of ATP from astrocytes. Recent data have implicated the adrenergic signaling in responsiveness of astrocyte networks to the behavioural state and sensory inputs [75, 76]. On the other hand, adrenergic neurons of *locus coeruleus*, the main source of noradrenaline release in the brain cortex, can undergo massive loss during neurodegenerative diseases [104]. Also, the release of ATP from astrocytes, including the one triggered by *α*1-adrenoreceptors, can decline with ageing [39]. Thus, astroglial release of ATP by astrocytes as well as by microglia and purinergic modulation of synaptic transmission may be of particular importance for the brain metaplasticity induced by experience, environmental factors, ageing, or neurodegenerative disease [6, 96, 105]. Conversely, massive release of ATP occurs also from damaged or dying cells after brain ischemia or trauma or during neurodegenerative diseases [1, 10, 38, 98] and is often correlated to increased expression of P2X receptors [1]. Exacerbating ATP P2X signaling may also have profound effect on brain synaptic activity or plasticity. These very interesting and timely topics are yet to be explored.

To conclude, recent results strongly support the physiological importance of P2X receptors and astroglial release of ATP. The purinoreceptor-mediated communication between astrocytes and neurons is essential for the regulation of synaptic strength and the modulation of synaptic plasticity.
